# Composition and Diversity Analysis of the Gut Bacterial Community of the Oriental Armyworm, *Mythimna separata,* Determined by Culture-Independent and Culture-Dependent Techniques

**DOI:** 10.1673/031.013.16501

**Published:** 2013-12-31

**Authors:** Cai He, Xiaoning Nan, Zhengqing Zhang, Menglou Li

**Affiliations:** 1 College of Forestry, Northwest A&F University, Yangling, Shaanxi, 712100, P. R. China; 2 Wuwei Academy of Forestry Sciences, Wuwei, Gansu, 733000, P.R. China

**Keywords:** 16S rDNA, intestinal bacteria, PCR-DGGE

## Abstract

The intestinal bacteria community structure and diversity of the Oriental armyworm, *Mythimna separata* (Walker) (Lepidoptera: Noctuidae), was studied by analysis of a 16S rDNA clone library, denaturing gradient gel electrophoresis,and culture-dependent techniques. The 16S rDNA clone library revealed a bacterial community diversity comprising Cyanobacteria, Firmicutes, Actinobacteria, Gracilicutes and Proteobacteria, among which *Escherichia coli* (Migula) (Enterobacteriales: Enterobacteriaceae) was the dominant bacteria. The intestinal bacteria isolated by PCR-denaturing gradient gel electrophoresis were classified to Firmicutes, Proteobacteria, and Gracilicutes, and *E. coli* was again the dominant bacteria. The culture-dependent technique showed that the intestinal bacteria belonged to Firmicutes and Actinobacteria, and *Staphylococcus* was the dominant bacteria. The intestinal bacteria of *M. separata* were widely distributed among the groups Cyanobacteria, Firmicutes, Actinobacteria, Gracilicutes, Proteobacteria, and Gracilicutes. 16S rDNA clone library, denaturing gradient gel electrophoresis, and culture-dependent techniques should be integrated to obtain precise results in terms of the microbial community and its diversity.

## Introduction

The Oriental armyworm, *Mythimna separata* (Walker) (Lepidoptera: Noctuidae), is a typical seasonal migrating pest in China that causes heavy losses to agricultural production and food security (Chen et al. 1995; Lee et al. 1995). For efficient control of *M. separata,* a lot of research on their biological and ecological habits has been performed, but little has been done relative to its population genetics and molecular ecology (Zhang and Zhai 2008). *Mythimna separata* is widespread in China and other countries and has been used as a test insect to explore new agricultural pesticides. For instance, the bioactivity of anthaddin (Zhang et al. 2003), coriaria lactone (Guo et al. 2007), tutin (Zhuang et al. 2007), *Datura stramonium* (Zhou et al. 2008), and celangulin V (Lü et al. 2008) have been measured based on *M. separata.* Recently, molecular biological approaches to the study of insect intestinal bacteria have received a great deal of attention, including studies on *Reticulitermes speratus* (Hongoh et al. 2003), *Ixodes ricinus* (Claudia et al. 2003), *Apriona germari* (Zhang et al. 2004), *Hepialus gonggaensis* (Liu et al. 2001; Zhuo et al. 2005), *Bombyx mori* (Xiang et al. 2007), and *Apis mellifera, Vespula vulgaris, Vespa crabro,* and *Nauphoeta cinerea oliv* (Mrazek et al. 2008). The insect guts may represent a large source of microbial diversity. These bacteria utilize a wide range of organic polymers and can be involved in methanogenesis and nitrogen fixation (Nardi et al. 2002). The gut microflora also play an important role in pheromone production, pesticide degradation, vitamin synthesis, and pathogen prevention (Reeson et al. 2003). In addition, these bacteria also take part in resisting the invasion and propagation of microbes and strengthening the function of the immune system (Dillon et al. 1995, 2002a, 2002b; Tokuda et al. 1997). Therefore, analysis of the composition and diversity of the intestinal bacteria of *M. separata* have application value in pest biocontrol and new pesticide exploration.

Bacterial communities in insect guts have been studied mainly by the cultivationdependent technique, which does not reflect the entire microbial communities (Gilliam et al. 1997). 16S rDNA genes, which exist in all nuclear biological cells, have been widely used to estimate the diversity of the insect gut bacterial microbiota (Reeson et al. 2003; Broderick et al. 2004). However, little information is available on the gut bacterial communities of many insects, including *M. separata.* In our study, a detailed analysis of bacterial diversity and community structure in the gut of the *M. separata* is reported, through the construction of the 16S rDNA clone library, PCR denaturing gradient gel electrophoresis (DGGE), and culturedependent techniques. The objective of this work was to demonstrate the bacterial community structure and diversity in *M. separata,* which would help to provide a novel method of pest biocontrol and to explore potential pesticides as well.

## Materials and Methods

### Sample collection

The *M. separata* were purchased from Northwest A&F University biorational pesticide research center, China. Corn leaves were used for food, and the insects were maintained at 22 ± 1° C. The isolated guts of 10 starved *M. separata* were put on ice and gently crushed using a pestle in liquid nitrogen. The entire experiment was repeated three times.

### DNA extraction and 16S rDNA amplification

The DNA extraction of culture bacteria and intestinal total bacteria was performed according to the procedure described by Yuan et al. (2009). The quality of genomic DNA was analyzed on 1.0% agarose gel, followed by staining with ethidium bromide.

The universal primers 27mf (5′- AGA GTT TGA TCM TGG CTC AG -3′) and 1492r (5′- ATG GGY TAC CTT GTT ACG ACT T -3′) (Weisburg et al. 1991) were used to amplify the full-length 16S rDNA gene of the culturable bacteria. 357f-GC (5- GCclamp-CCT ACG GGA GGC AGC AG -3) and 517r (5′- ATT ACC GCG GCT GCT GG -3′) were used to amplify thel6S rDNA V3 region for DGGE, 341F (5′- CCT ACG CGA GGC AGC AG -3′) and 517r (5′- ATT ACC GCG GCT GCT GG -3′) (Muyzer et al. 1993) for the construction of a cloning library. The amplification was performed in a 50 µL reaction mixture consisting of 25 µL 2x ES Taq MasterMix (CWBio, www.cwbiotech.com), 2 µL each primer, 2 µL genomic DNA, and 19 µL RNA-free water. The PCR reaction was performed in a DNA Engine Dyad Peltier Thermal Cycler (Bio-Rad, www.bio-rad.com). The PCR amplification conditions were as follows: predenaturing at 94° C for 5 min, followed by 29 cycles of 94° C for 1 min, 55° C for 1 min, 72° C for 1.5 min, and a final extension at 72° C for 10 min. Finally, the amplification product was purified using a universal DNA purification kit (TIANGEN, www.tiangen.com).

### Isolation and culture of the intestinal bacteria

Guts of ten healthy M *separata* larvae were homogenized in 1 mL sterilized distilled water, and then serial 8-fold dilutions were made, also using sterilized distilled water. Aliquots (0.1 mL) of the 10^-3^ to 10^-5^ dilutions were spread onto conventional agar plates (potato dextrose agar, nutrient agar medium, beef-extract-peptone-dextrose, Miller and Schroth medium, and glucose ammonium salt culture medium. The plates were cultivated for 48 hr at 37° C. Selected different bacteria strains were pure cultured for 48 hr at 37° C, then extracted DNA from pure cultured strains was selected for PCR and sequencing.

### Construction of 16S rDNA library

The intestinal total bacteria DNA of M *separata* was amplified and purified according to the above DNA extraction and amplification process. PCR products were cloned into pUCm-T according to the manufacturer's instructions (Sangon, www.sangon.com) and then transported into competent cells (SK2301, Sangon). Plasmid insertions were screened following the manufacturer's instructions. Segment size was checked by PstI single enzyme cut and agarose gel electrophoresis to confirm whether the target fragment was present or not. The cloning vector was sequenced by the universal primer ml3.

### DGGE analysis

DGGE was performed with the Dcodek Universal Mutation Detection System (Bio-Rad), according to the manufacturer instructions. DGGE of the amplified 16S rDNA gene was performed with 8% acrylamide gel containing a denaturant gradient of 35–60% (100% defined as urea 7 M, 40% deionized formamide). The total of PCR products was concentrated to a 30 µL final volume and loaded on gel, which was electrophoresed at a constant voltage of 80 V for 1 hr, 60 V for 16 hr, and 60° C in 1 TAE buffer (40 mM Tris, 20 mM acetate, 1.0 mM Na2-EDTA). After electrophoresis, DGGE gel was stained with ethidium bromide and photographed with UV transillumination. Then, characteristic bands were excised from the gel and dissolved with sterilized distilled water at 4° C for a night. The supernatant after centrifugation (10000 rpm, 5 min, 4° C) was used as a DNA template for 16S rDNA-V3 amplification using the same primers without a GC-clamp. The PCR products were analyzed by electrophoresis in 1.5% agarose gel. Finally, the amplification products were purified using a universal DNA purification kit and sequenced.

### Phylogenetic sequence analysis

The sequences were compared with known sequences listed in the GenBank nucleotide sequence databases. The BLAST search option of the National Center for Biotechnology Information (NCBI) (GenBank ID: BA123456) (http://www.ncbi.nlm.nih.gov) was used to search for close evolutionary relatives in the GenBank database (Altschul et al. 1997). A neighbor-joining tree of the aligned sequences was constructed using MEGA 5.0 (Kumar et al. 2001).

**Figure 1.**
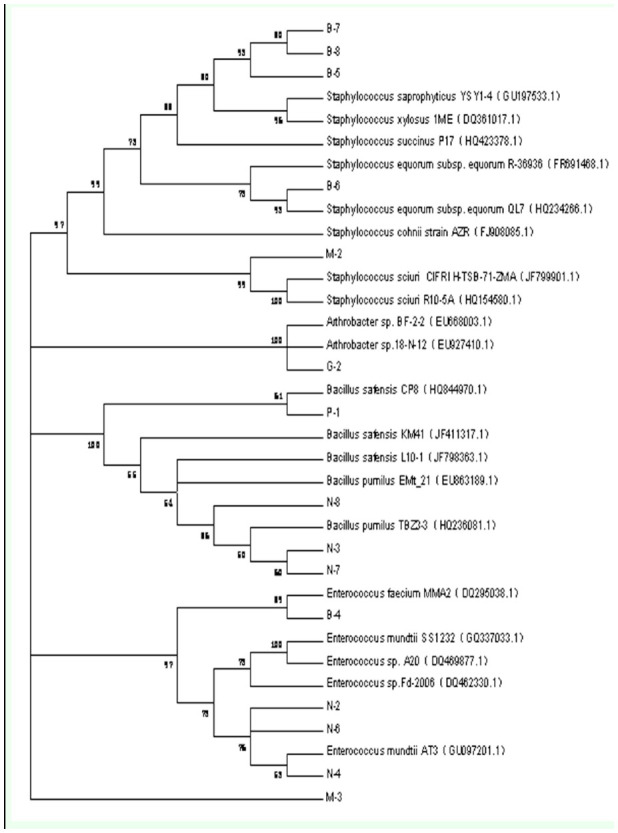
The phylogenetic tree of *Mythimna separata* intestinal bacteria obtained using the culture-dependent method. The following reference species were used: *Staphylococcus saprophyticus:* B-5, B-7, B-8; *Staphylococcus equorum:* B-6; *Staphylococcus sciuri:* M-2; *Arthrobacter.* G-2; *Bacillus pumilus:* N-3, N-7, N-8, M-3; *Enterococcus faecium:* B-4; *Enterococcus mundtii:* N-2, N-4, N-6; *Bacillus safensis:* P-. High quality figures are available online.

## Results

### Cultured bacterial community of *M. separata*

In our study, 30 bacterial strains were isolated from five different culture mediums, and 20 16S rDNA sequences were obtained with the BLAST search from the database of the National Center of Biotechnology Information. Four bacterial strain sequences were the same as the strain B-5, and one was the same as the N-4 strain, so these strains are not seen in Figure 1. The cultured bacterial sequences obtained in our study are available in the GenBank nucleic acid sequence database under the accession numbers JN995574–JN995588. The results of our study were compared the identities of these bacterial strain sequences to the reported sequences in the NCBI database, and they were over 99% similar, except M-3, which only had 97% similarity with *Bacillus pumilus* Meyer and Gottheil (Bacillales: Bacillaceae). Four different phylotypes were isolated. *Staphylococcus* was the dominant component (9/20, 45%). The other strains belonged to the genera *Bacillus, Arthrobacter,* and *Enterococcus.* The identities of these strain sequences reported in the NCBI database were over 99%. The neighbor-joining method of MEGA 5 was used to construct a phylogenetic tree (Figure 1). The results indicated that *Staphylococcus saprophyticus* (Fairbrother) (Bacillales: Staphylococcaceae) (7/9, 77.78%), *B. pu*milus* (3/4, 75%) and *Enterococcus mundtii* Collins (Lactobacillales: Enterococcaceae) (4/5, 80%) were the dominant species.*

**Table 1.**
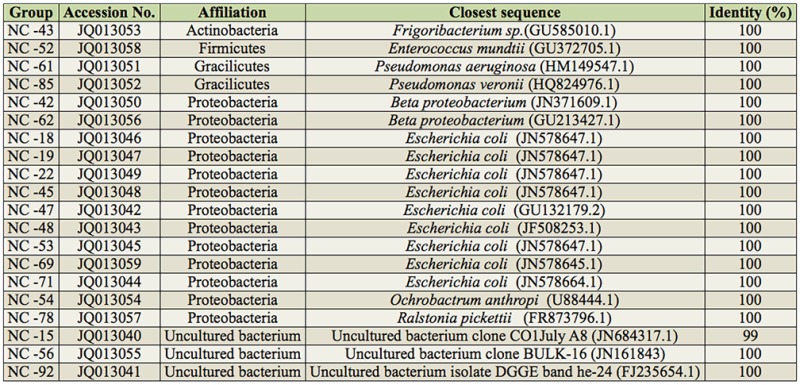
The intestinal bacteria 16S rDNA sequences identified by the clone library.

Different bacterial culture strains were isolated from five bacterial culture mediums. The results showed that more bacteria, including *Enterococcus, Bacillus, Staphylococcus,* were isolated from NA and Miller and Schroth media than other media. *Staphylococcus* and *Enterococcus* were isolated from the beef-extract-peptone-dextrose medium. *Arthrobacter* and *Bacillus* were isolated from both the glucose ammonium salt culture medium and the potato dextrose agar medium.

### Construction of 16S rDNA gene clone library

The 16S rDNA gene clone library consisted of 100 clones from *M. separata.* Twenty typical clones were selected to sequence. According to the sequences, intestinal bacteria were classified into four domains: Firmicutes, Actinobacteria, Gracilicutes, and Proteobacteria. Thirteen clones were clustered to Proteobacteria and nine clones of those were closely related to the genus *Escherichia.* Four clones of these had a high degree of similarity (100%) to the previously sequenced organisms *Ralstonia picket*tii (clone NC-78), *Ochrobactrum anthropi* (clone NC-54), and *Beta-proteobacteria* (clones NC-42 and NC-62). Firmicutes and Actinobacteria had one clone each, *Enterococcus mundtii* and *Frigoribacterium,* respectively. Three clones had high identities with uncultured bacterium sequences from the NCBI database (clones NC-56, NC-15, and NC-92) (Table 1). All the clone sequences obtained in our study have been assigned to the GenBank nucleic acid sequence database, with accession numbers JQ013040– JQ013059.

### DGGE pattern and phylogenetic analysis of the intestinal bacteria of *M. separata*

**Figure 2.**
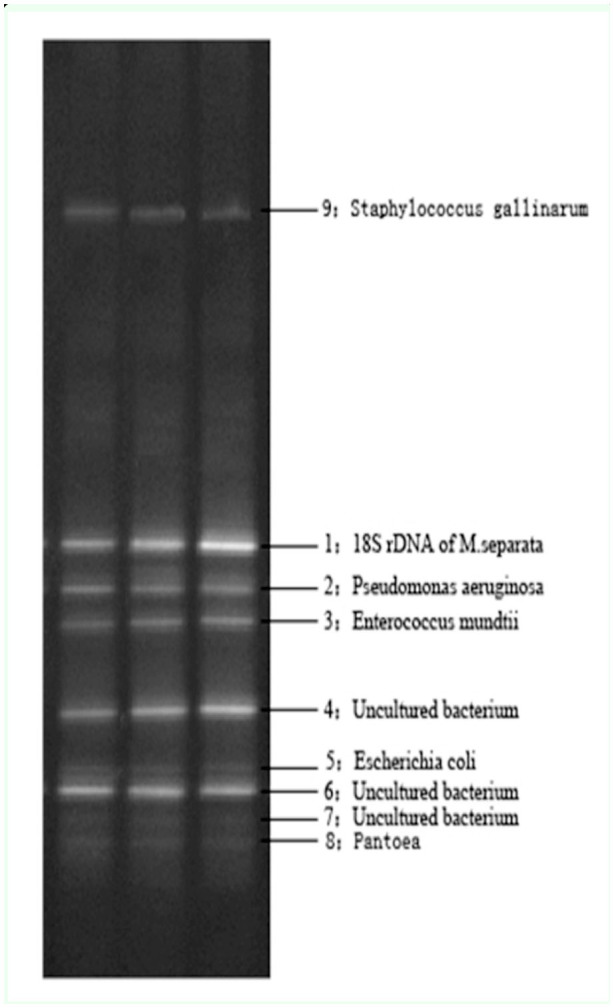
Detail of the ethidum-bromide-stained 16s rDNA DGGE fingerprints of *Mythimna separata* intestinal bacteria. The following reference species were used: *M. separata* (1), *Pseudomonas aeruginosa* (2), *Enterococcus mundtii* (3), *Escherichia coli* (5), *Pantoea* (8), *Staphylococcus gallinarum* (9), uncultured bacterium (4, 6, 7). Three electrophoresis lanes were the repetition of the sample. High quality figures are available online.

**Figure 3.**
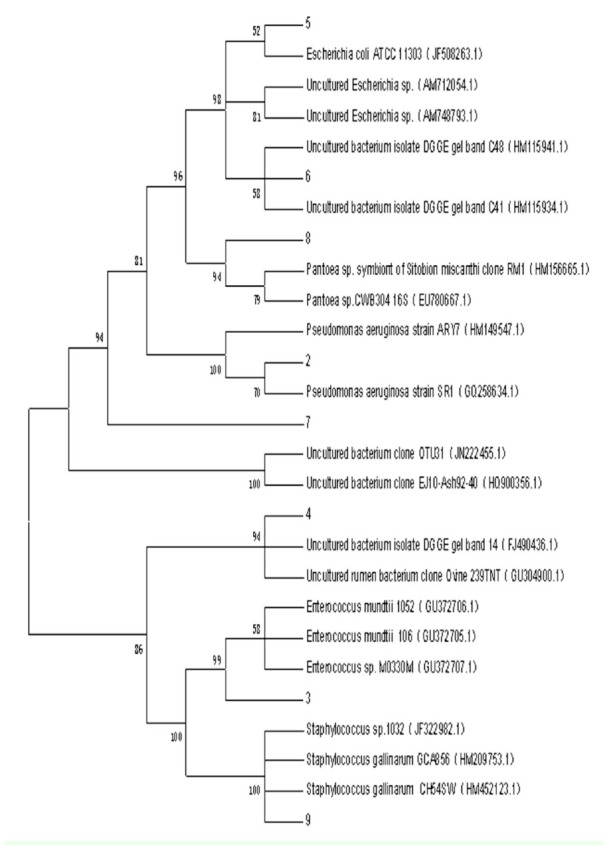
The phylogenetic tree of *Mythimna separata* intestinal bacteria obtained using DGGE. The following reference species were used: *M. separata* (1), *Pseudomonas aeruginosa* (2), *Enterococcus mundtii* (3), *Escherichia coli* (5), *Pantoea* (8), *Staphylococcus gallinarum* (9), uncultured bacterium (4, 6, 7). High quality figures are available online.

For phylogenetic identification, sequences were compared with 16S rDNA sequence information of known bacteria listed in the GenBank databases. The predominant DGGE bands of *M. separata* intestinal bacteria are shown in Figure 2, and the results of phylogenetic analysis are shown in Figure 3, revealing the presence of a variety of different genera. Eight bacterial sequences were divided into three main groups through neighbor joining analysis: Firmicutes, Proteobacteria, and Gracilicutes. Similarity values were between 99% and 100% to *Enterococcus* (band 3), *Pantoea* (band 8), *Pseudomonas* (band 2), *Escherichia* (band 5), and *Staphylococcus* (band 9). Band 7 was the most closely related (91%) to *Pseudomonas,* bands 4 and 6 had highly similar identities (100%) to uncultured bacterium sequences from the NCBI database (FJ490436.1 and HMI 15934.1). All the DGGE sequences obtained in this study have been assigned to the GenBank nucleic acid sequence database, with accession numbers JQ039351– JQ039358.

### Compared**** *M. separata* intestinal bacteria gained by different methods

Eleven known bacterial genera were isolated by three methods in this experiment. Table 2 shows that the construction gene library of bacterial species was detected to be the highest percentage, accounting for 72.72% (8/11). DGGE accounted for 45.45% (5/11), and culture-dependent accounted for 36.36% (4/11). The common bacteria determined by construction of the gene library, DGGE separation, and conventional culture was *Enterococcus. Bacillus, Arthrobacter,* and *Staphylococcus* were only isolated by culture-dependent methods, while *Escherichia, Beta proteobacterium, Frigoribacterium, Ochrobactrum, Pantoea, Pseudomonas,* and *Ralstonia* were detected by molecular biology methods (PCR-DGGE and construction of the gene library) (Table 2).

**Table 2.**
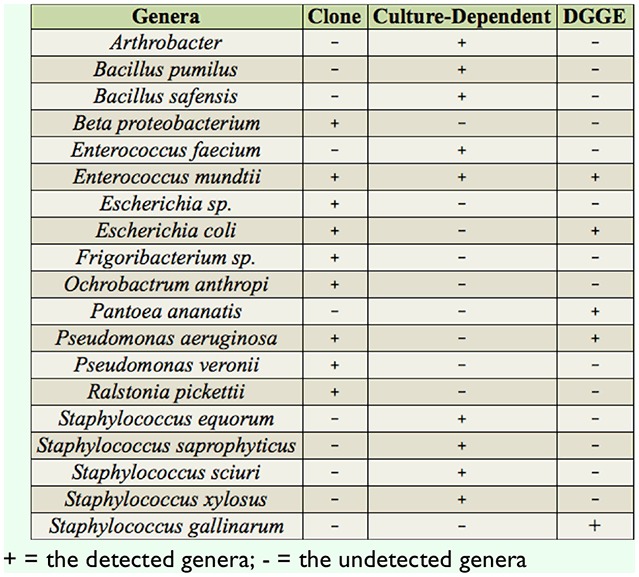
Comparison of intestinal bacteria gained by different methods.

## Discussion

The culture-independent nucleic acids-based techniques, such as DGGE and clone library techniques, were used to reveal the intestinal bacteria community structure of *M. separata.* Eleven genera of intestinal bacteria in *M. separata* were isolated in this study. Different intestinal bacteria were obtained by three methods. Four genera of intestinal bacteria were gained by culture-dependent techniques, among which *Staphylococcus* was the predominant bacteria. *Escherichia* was the predominant genus among the six genera of intestinal bacteria isolated by constructing the clone library and the five genera of intestinal bacteria isolated by DGGE. The intestinal bacteria of *M. separata* obtained from construction of the gene library were greater in number than the number obtained using the DGGE and culture-dependent methods, indicating that the construction of the gene library revealed higher bacterial diversity than the DGGE and culturedependent methods. Thus, the construction of a gene library could identify the most bacteria strains, while DGGE isolated the predominant bacteria, and the culturedependent method was useful for identifying a few bacteria. Using DGGE or a culturedependent technique alone reduces the integrity of the experimental results and results in a lower diversity of bacteria. Moreover, the three methods showed different predominant bacteria. The difference in results is because the culture-dependent technique inevitably screens for microorganisms. For instance, some of the bacteria present in low amounts would grow rapidly and become the dominant bacteria in a short time, while some highly abundant bacteria could not be identified, because the artificial separation culture conditions are not suitable for their growth. In addition, most of the highly abundant bacteria gained by molecular biology methods cannot be isolated or cultured by traditional bacteria culture methods, and bacteria isolated by culture-dependent techniques often exhibited low abundance when molecular biology methods are used.

DGGE band 1 was identified as 18S rDNA of *M. separata.* Comparative sequence analysis revealed that the universal primer 517r completely annealed to the *M. separata* 18S rDNA. Although primer 341f is a bacterial-specific 16S rDNA primer, and comparison to the *M. separata* 18S rDNA sequence revealed four unannealed base pairs, 18S rDNA was amplified with the primer set 341f and 517r under the given PCR conditions (Claudia et al. 2003). Nested PCR and touch down PCR amplification could increase the specificity of the 16S rDNA amplification and circumvent this problem.

Most of the bacteria that were isolated in this study have been found in other insects. Species of the genus *Staphylococcus,* which are gram-positive bacteria and typically unpathogenic, can often be found in the gut of insects. Eutick et al. (1978) reported the intestinal bacteria of 10 kinds Australia termites and found that *Staphylococcus* was the predominant bacteria. *Staphylococcus* was also isolated from the gut of *Apriona germari* with traditional culture-dependent techniques (Zhang et al. 2004), from *Hepialus gonggaensis* by culture-dependent and culture-independent techniques (Zhuo et al. 2005), and from *Bombyx mori* using library sequence analysis (Xiang et al., 2007). Therefore, *Staphylococcus* is the normal intestinal bacteria of many insects. From the point of view of animal micro-ecology, the nature, amount, and location conditions of the normal gut microflora may have a very close relationship to insect nutrition and digestive physiology (He et al. 1994). Lots of research has been done on the mechanism of action in *Bacillus, Pseudomonas, Escherichia.,* and *Enterococcus.* Mead et al. (1988) studied one kind of grasshopper (*Melanoplus sanguinipe*) intestinal *Enterococcus* and found that many *Enterococcus* species could produce acetate, and speculated that the accumulation of a large amount of acetate reduced the intestinal pH of the grasshopper. Broderick et al. (2004) surveyed the intestinal bacteria of *Lymantria dispar* larvae and found that *Enterococcus* was the dominant bacterium; furthermore, the authors suggested that reducing intestinal pH could protect the host from toxins. Prescott et al. (2002) found that the common characters of *Pseudomonas* and *Escherichia* species were organic nutrition and facultative anaerobes, and that they could degrade carbohydrates via glycolysis and the pentose phosphate pathway.

Yang et al. (2010) found *Bacillus, Pseudomonas, Pantoea,* and *Proteus* in the gut of *Apis mellifera ligustica.* Both He et al. (2001) and Zhang et al. (2004) found *Staphylococcus, Bacillus, Escherichia,* and *Proteus* in the gut of *Apriona germari.* Broderick et al. (2004) found *Bacillus, Pseudomonas, Pantoea, Staphylococcus,* and *Enterococcus* in the gut of *Lymantria dispar.* So, we speculated that the gut bacterial community composition of M *separata* would have many similarities with insects of Hymenoptera, Coleoptera, and Lepidoptera. These insects have different living conditions, habits and food, but *Bacillus, Pseudomonas, Pantoea, Staphylococcus, Enterococcus, Proteus,* and *Escherichia* were found in M *separata* guts. This indicated that these intestinal bacteria have no relationship with feeding methods and that they are the intrinsic gut bacteria of insects.

As shown in our study, three methods were used to identify bacterial diversity of *M. separata.* Nevertheless, as with each method, the applied approach is not free from bias. Limitations such as preferential amplification of 16S rDNA of some bacterial taxons (Farrelly et al. 1995; Polz and Cavanaugh 1998) and identical electrophoretic migration of sequences with multiple differences lead to an underestimation of bacterial diversity in DGGE community fingerprints (Jackson et al. 2000). In addition, DGGE bands have short sequences (about 150 bp in the present study, which may have affected the resolution of the taxa on the plots and may limit the available analysis (Asakawa and Kimura 2008)). Clone library analysis can identify more bacterial strains, but are not suitable for handling multiple samples due to heavy workload and high cost. Culture-dependent methods cannot reveal bacterial community effectively, because most of bacteria have strict living conditions. Therefore, culture-independent and culture-dependent methods should be combined so that the composition of microbial community structure and diversity can be better revealed.

## Abbreviations

DGGE,: denaturing gradient gel electrophoresis
